# A Rare Case of Acquired Bleeding Disorder in a 24-Year-Old Hispanic Female

**DOI:** 10.7759/cureus.25780

**Published:** 2022-06-09

**Authors:** Ayrton I Bangolo, Mahabuba Akhter, Jeffin P Cherian, Bhavna Gupta, Sameh Elias

**Affiliations:** 1 Internal Medicine, Palisades Medical Center, North Bergen, USA; 2 Biology, Southern Arkansas University, Magnolia, USA; 3 Hematology and Oncology, Palisades Medical Center, North Bergen, USA; 4 Internal Medicine, HMH Palisades Medical Center, North Bergen, USA

**Keywords:** nutrition, coagulation, dietary insufficiency, vitamin k, menorrhagia, bleeding

## Abstract

Vitamin K is a fat-soluble vitamin that has a major role in coagulation pathways thus its deficiency can lead to major bleeding disorders. Vitamin K deficiency in an otherwise healthy adult is rare. Inadequate oral intake of vitamin K remains the most common cause of vitamin K deficiency. Here, we report a young female who presented for evaluation of heavy menstrual bleeding and was found to be deficient in vitamin K-dependent coagulation factors, with correction upon mixing study. She was diagnosed with vitamin K deficiency due to poor oral intake. With this case, we report a rare and avertible cause of major bleeding to raise awareness among clinicians about patients’ daily nutritional requirements.

## Introduction

Vitamin K deficiency in an otherwise healthy adult is rare. This is largely due to the wide distribution of phylloquinone in plants, menaquinone production by gut microflora, and because vitamin K is easily recycled within cells [1]. Vitamin K is a fat-soluble vitamin that can become deficient in any malabsorption state [1]. The most common cause of vitamin K deficiency is poor oral intake [2]. Bleeding is the major symptom of vitamin K deficiency, with involvement of any site [3]. Here we report the case of a 24-year-old female who presented with excessive menstrual bleeding due to a deficiency of vitamin K-dependent coagulation factors. Herein we portray the importance of educating patients on their daily nutritional requirements.

## Case presentation

This is a 24-year-old female with no significant past medical history who presented for evaluation of heavy menstrual bleeding associated with clots, hematuria, and abdominal cramping. She also reported shortness of breath, dizziness, and fatigue. The patient reported a significant increase in the number of menstrual pads used. She denied having a similar problem in the past since her menarche which occurred at age 14. She noticed diffused bruising, red spots in the buccal mucosa, and epistaxis a few days before the onset of heavy vaginal bleeding. She recently started a new job as an industrial machine operator and for the past six months, she has only been eating fast food. The patient’s diet mainly consists of carbohydrates, rice, beans, and plantains; she has not eaten vegetables or fruits for many years. 

On physical examination, she was found to be tachycardic, there was conjunctival and skin pallor, oral mucosal petechial hemorrhage, extensive bruising of the upper extremities, and heavy vaginal bleeding with visible clots. Laboratory results revealed a deficiency of vitamin K-dependent factors with improvement upon mixing study as seen in Table 1.

**Table 1:** Lab values before and upon mixing

	Lab values	Lab values upon mixing
Partial thromboplastin time (PTT)	84.6 seconds (26.0-36.0)	44.9 seconds
Prothrombin time (PT)	> 320 seconds (9.5-12.8)	3.86 seconds
International normalized ratio	> 13.30 (0.85-1.13)	3.86 seconds
Activated clotting time of thromboelastography (T-ACT)	238 seconds (86-118)	
Factor II activity	9.9% (73-138)	32%
Factor VII	9% (60-150)	27%
Factor IX	53% (60-160)	63%
Factor X	4% (70-150)	36%
Hemoglobin	6 gram per deciliter (mg/dl) (12-16)	

It also revealed low hemoglobin and normal liver function tests. A pregnancy test was negative. A pelvic ultrasound revealed a normal-appearing uterus and ovaries with an endometrial stripe measuring 0.7 cm. Based on the laboratory results and the patient’s history, a diagnosis of vitamin K deficiency was made.

She received four units of pure red blood cells, vitamin K intravenously, medroxyprogesterone, and fresh frozen plasma. Hematology, gynecology, and dietetic consultations were obtained. The patient was educated on dietary sources of vitamin K. The patient’s bleeding was controlled on the sixth day of hospitalization, and she was discharged on an oral vitamin K supplement.

## Discussion

Vitamin K deficiency can occur in any age group but is encountered most often in infancy. It is an essential, lipid-soluble vitamin that plays a vital role in the production of coagulation proteins, and is found in green, leafy vegetables, and oils, such as soybean, cottonseed, canola, and olive oils. Vitamin K is also synthesized by colonic bacteria [3]. Deficient dietary intake, malabsorption, and decreased production of intestinal bacteria (due to treatment with chemotherapy or antibiotics) are common causes of VK deficiency [4]. The most common cause is poor oral intake [2]. Our patient’s diet was mainly consistent with carbohydrates known to have low vitamin K content such as rice, beans, and plantains [5]. She did not include leafy vegetables for many years in her diet. She was counseled on the importance of incorporating more vitamin K-rich food in her diet to maintain a daily intake of 90 micrograms [5].

Prolongation of prothrombin time (PT) values is the most common and earliest finding in vitamin K-deficient patients [6]. As with liver disease, patients with vitamin K deficiency have PT levels disproportionately longer than partial thromboplastin time (PTT) levels [2]. Thus, mild to moderate vitamin K deficiency typically only features a prolonged PT that corrects upon mixing [7]. As seen in our patient, the PT level was significantly increased as compared to the PTT level, and PT, PTT, and other vitamin K-dependent factors were corrected upon mixing, consistent with a diagnosis of vitamin K deficiency. 

Vitamin K depletion can occur in as little as two weeks if both intake and endogenous production of vitamin K are eliminated [8]. It can take longer if only the dietary intake is at fault, such as in our patient. Parenteral administration of vitamin K at a total dose of 10 mg is sufficient to restore normal levels of clotting factors within eight to 10 hours. In the presence of ongoing bleeding, replacement with fresh frozen plasma or prothrombin concentrate is required [6]. Our patient received vitamin K intravenously and fresh frozen plasma was administered given the ongoing bleeding.

Our patient was not recently exposed to antibiotics that could have disturbed the gut flora, and nor did she have clinical findings or a history of a malabsorptive state. She was unaware that her diet was not meeting the minimum requirement for daily vitamin K supplementation.

## Conclusions

Vitamin K is a fat-soluble vitamin with a major role in coagulation pathways. Vitamin K deficiency is a rare occurrence in adults and can lead to life-threatening major bleeding. Poor dietary intake is the most common cause and in the absence of a malabsorptive state, it can take several months to years for the patient to develop major bleeding. With this case report, we hope to encourage physicians to educate patients about their daily nutritional requirements. 
